# Urban mobility and crime: causal inference using street closures as an instrumental variable

**DOI:** 10.3389/fdata.2025.1579332

**Published:** 2025-10-31

**Authors:** Karl Vachuska

**Affiliations:** Department of Sociology, University of Wisconsin-Madison, Madison, WI, United States

**Keywords:** everyday mobility patterns, crime, visitors, neighborhoods, causal inference

## Abstract

The advent of widely available cell phone mobility data in the United States has rapidly expanded the study of everyday mobility patterns in social science research. A wide range of existing literature finds ambient population (e.g., visitors) estimates of an area to be predictive of crime. Much of the past research frames neighborhood visitor flows in predictive terms without necessarily indicating or implying a causal effect. Through the use of two causal inference approaches—conventional two-way fixed effects and a novel instrumental variable approach, this brief research report explicitly formulates the causal effect of visitors in counterfactual terms. This study addresses this gap by explicitly estimating the causal effect of visitor flows on crime rates. Using high-resolution mobility and crime data from New York City for the year 2019, I estimate the additive effect of visitors on the multiple measurements of criminal activity. While two-way fixed effects models show a significant effect of visitors on a wide array of crime forms, instrumental variable estimates indicate no statistically significant causal impact, with large standard errors indicating substantial uncertainty in visitors' effect on crime rates.

## Introduction

Recent advances in the widespread availability of data on everyday mobility patterns have substantially reshaped the geospatial predictive modeling of crime (Vachuska, 2022). While the population offset for studying crime has historically been restricted to residential population counts, ambient population estimates have proven to be a better predictor for a number of reasons (Rummens et al., 2021). Mobile phone data improve crime rate estimations and spatiotemporal predictions as they more closely reflect real-time population densities in various locations (Rummens et al., 2021). Hanaoka (2016) highlights that mobile phone data offers a more precise spatial and temporal resolution compared to traditional datasets, thereby enhancing the understanding of urban activities and their correlation with crime. The continuous tracking of mobile phone users allows researchers to analyze how fluctuations in population density correlate with crime occurrences, providing critical insights into when and where crimes are likely to happen.

Extensive research has explored the relationship between mobility patterns and crime. Schertz et al. (2021) illustrate how mobility data can be incorporated into evaluations of neighborhood characteristics that are critical for crime prediction. Broadly, scholars have argued that mobility data can enhance the prediction of criminal activity—particularly for rare or underreported events that might be misestimated otherwise (Wu et al., 2020). Additionally, mobility data can be used to aid in the identification of potential crime hotspots and the likelihood of criminal activities occurring in specific areas (Song et al., 2019; Kadar and Pletikosa, 2018), substantailly enhancing the predictive capabilities of crime models. As a result of these uses, mobile phone data has been increasingly popular in predictive policing (Goerzig, 2016). Ultimately, models based on aggregated mobile phone data have remarkably high accuracy in forecasting future criminal activity (Bogomolov et al., 2015).

While research has disproportionately focused on the mobility flows for predictive analyses, the underlying nature of the association is more convoluted. Haleem et al. (2020) emphasizes the concept of the “exposed population,” particularly in nightlife contexts, arguing that fluctuations in ambient population alter crime opportunities in public spaces. Haleem et al. (2020) argues that visitor flows influence crime patterns by altering the number of potential offenders, victims, and guardians at a given location. While not making a causal argument, Haleem and colleagues contribute to a theoretical perspective on how visitor flows may be a causal variable by grounding mobility in the context of routine activities theory. While the use of everyday mobility patterns for predictive policing has substantial ethical issues, I argue that a better understanding of the causal effect of visitor flows can inform an understanding of criminal activity that can shape equitable and ethical policies aimed at reducing crime.

Disproportionately, research looking at the impact of tourism on criminal activity has better situated visitor flows as a causal variable rather than simply a predictive one. Melo et al. (2017) argue that tourists have a unique impact on crime rates, as visitors may increase the number of potential targets for criminal activities at higher rate than residents themselves might. The presence of tourists can create a higher density of individuals who are less familiar with their surroundings, making them more vulnerable to crime (Montolio and Planells-Struse, 2016). Indeed, empirical research has found that tourism is positively correlated with some types of crime. For instance, Montolio and Planells-Struse (2016) found that in top tourist destinations, there is a significant increase in both property crimes and violent crimes, suggesting that the influx of visitors can create opportunities for criminal behavior. Similarly, Sypion-Dutkowska and Dutkowski (2021) found a strong correlation between tourism density and crime rates, indicating that areas with high tourist activity often experience higher crime rates, though specifically property crimes. Similarly, Zhang and Xiang (2021) utilized a dynamic spatial model to demonstrate that tourism development can lead to increased crime rates in certain contexts, although they also noted that tourism could have a mitigating effect on crime in other scenarios.

In distinction, some studies actually suggest that tourism can have a mitigating effect on crime. For example, Zhang and Xiang (2021) noted that in some cases, tourism can exert a negative impact on crime, potentially due to increased economic activity and community engagement that discourage criminal behavior. Similarly, Altindag (2014) found that while increased crime can deter tourism, a thriving tourism sector can also contribute to local economic prosperity, which may indirectly reduce crime rates in the long term. This suggests that while much research has found a larger ambient population size to be associated with more crime, there are potentially theoretical pathways by which visitors could actually *reduce* crime.

Social disorganization theory also theoretically informs how visitor flows may impact crime rates. High levels of visitors may reduce the social cohesion within an area, as residents are less likely to be familiar with visitors. Effendi (2023) highlights that as the number of tourists rises, the social cohesion of local communities may weaken, creating an environment conducive to crime. This notion would imply that neighborhoods with high visitor turnover may struggle to maintain social order, leading to increased opportunities for criminal activity. This theory, however, to some extent suggests that this breakdown of social order would take place over a long period of time and thus might not be detectable in analyses that can only consider short-term effects.

Ultimately, in spite of a wide-array of past research highlighting a strong descriptive association between visitor flows and crime, very little prior research has engaged with visitor flows as a causal variable. This is likely attributable to endogeneity concerns making it difficult to identify causal effects using observational data. In particular, unobserved neighborhood attributes, or even reverse causality complicates identification. In this paper, I aim to address this gap by formulating the effect of visitor volume on neighborhood crime using a counterfactual formulation, thereby providing causal estimates of the effect of visitor volume on neighborhood crime. I use street closures as a novel instrumental variable, exploiting these exogenous shocks to local mobility patterns that are plausibly unrelated to crime trends other than through mobility flows. By integrating high-resolution cell phone mobility data and crime reports at the census block group–day level across all of New York City, I test whether increased visitor flows cause higher crime rates. Ultimately, this work contributes to the literature by explicitly addressing endogeneity concerns, demonstrating the utility of street closures as an instrument for visitor flows, and informing policy debates on crime prevention in high-traffic urban areas.

## Data

This study utilizes multiple data sources to construct a panel dataset at the census block group-day level for all of 2019 in New York City. The primary datasets include street closure records, crime reports, and mobility data.

I use data from the “Street Closures due to construction activities by Intersection” dataset, which records street closures throughout New York City in 2019. Each closure is geocoded and linked to the geodesically closest census block group by matching the closure's location to the centroid of the nearest census block group. This allows me to construct a daily indicator variable for whether at least one street closure occurred in each census block group.

Crime data comes from the New York City Police Department's Historic Complaint Data dataset, which includes all reported felony, misdemeanor, and violation-level crimes from 2006 through 2019. I extracted crime reports for all of 2019 and geocoded each reported offense to its corresponding census block group based on the reported incident location using the point-in-polygon method. I focus on seven crime outcomes:

- Petit larceny
- Assault (misdemeanor level)
- Felony assault
- Murder
- Impaired driving
- Traffic violations
- All crimes (any reported offense)

Given the low frequency of individual crime types in any census block group on a given day, I construct binary indicators for each crime outcome, coded as 1 if at least one such crime occurred in a census block group on a given day, and 0 otherwise.

I measure daily visitor totals for each census block group using SafeGraph's social distancing metrics dataset, which provides anonymized mobility data based on GPS pings from mobile devices. This dataset allows for the estimation of the number of non-residents entering each census block group per day.

I estimated the number of visitors between each pair of census block groups in the United States for each day in 2019 according to the following formula:


(1) $$\begin{array}{l} V_{ijd}=\;\frac{v_{ijd}\;*\;p_{i}}{n_{id}} \end{array}$$

where *V*_*ijd*_ is the estimated total number of daily visitors from block *i* to block *j* summed over all days in 2019, *v*_*ijd*_ is the number of visitors from *i* to *j* on day *d*, *p*_*i*_ is the estimated residential population in block *i* based on the 2015-2019 ACS estimates, and *n*_*id*_ is the number of SafeGraph panel devices whose home location was in block *i* on day *d*. One limitation of the SafeGraph data is the potential for visitor counts to be biased/inaccurately measured. The weighting procedure adjusts for the count of devices across census block groups varying by day, ensuring that each census block groups' device count reflects it's true population. However, if the panel of devices are not representative that may induce bias in the visit count estimates. Some past research does suggest some bias in the SafeGraph sample (Li et al., 2024). However, other research has also noted that the overall panel is about what one would expect from a large-N random sample (Noi et al., 2022). Additionally, other research suggests that for large-scale analyses, sampling errors tend to even out, suggesting any existing bias is minimal (Brazil et al., 2024).

Using these data sources, I construct a panel dataset at the census block group-day level for all of 2019. Each observation corresponds to a specific census block group on a specific day, containing information on whether a street closure occurred, the number of visitors, and the presence or absence of each crime type.

## Methods

The main method I employ to estimate the causal effect of the number of visitors to a neighborhood on crime rates is instrumental variable regression, one of the most popular methods for estimating causal effects using observational data (Angrist and Krueger, 1991; Angrist and Imbens, 1995; Angrist et al., 1996; Angrist and Pischke, 2009; Imbens and Rubin, 2015; Wooldridge, 2010; Morgan and Winship, 2014). The instrumental variable I utilize is a binary indicator for whether there was at least one street closure in the neighborhood on a given day. The street closure variable is determined using data from all census block groups in New York City that experienced at least one street closure in 2019. Geographical units are defined as census block groups, and I analyze data across all 365 days of 2019.

The key assumptions involved in this instrumental variable approach are relevance, exogeneity, and exclusion restriction. The relevance assumption requires that street closures are associated with the number of visitors to a neighborhood. To confirm this, I conduct a first-stage regression, which indicates a strong and statistically significant relationship between street closures and the number of visitors. The F-value for this regression exceeds the conventional threshold for a strong instrument. The exogeneity assumption requires that the occurrence of street closures is uncorrelated with unmeasured determinants of neighborhood crime rates. While this assumption is untestable, the inclusion of day-of-year and neighborhood fixed effects helps mitigate potential biases, and there is little reason to believe that street closures would not be exogenous net of these factors. The exclusion restriction assumption requires that street closures affect crime rates only through their effect on the number of visitors to the neighborhood. This assumption is also untestable, but there is no research to the best of my knowledge that suggests other ways that street closures might impact crime rates.

In addition to the instrumental variable, I incorporate fixed effects to control for potential confounders. Specifically, I use two-way fixed effects for neighborhoods and days of the year, which account for time-invariant differences across neighborhoods and seasonal or day-specific patterns in crime rates. Through the inclusion of these fixed-effect terms, I reduce potential bias from omitted variables that vary across neighborhoods or time and may potentially lead to a violation of the exogeneity assumption.

To estimate the causal effect, I compare the results from the instrumental variable regression with those obtained from a two-way fixed effects ordinary least squares (OLS) regression under the standard “no unobserved confounding” assumption. The conventional OLS model can be written as:


(2) $$\begin{array}{l} CRIME_{ij}=\beta_{0}+\beta_{1}\;*\;\log\left(visitors\right)+\Delta_{i}+\nabla_{j}+\epsilon \; \end{array}$$

Here, *CRIME*_*ij*_ represents the dichotomous presence of crime in a neighborhood *i* on day *j*, log(visitors) is the logged count of the number of visitors to the neighborhood, Δ_*i*_ represents census block group-fixed effects and ∇_*j*_ represents day-fixed effects, and ϵ is an error term with standard statistical assumptions. The estimate of β_1_ represents the OLS estimate of the association between visitors and crime rates, which may be biased to the extent that the unobserved confounding assumption is unreasonable.

To address the potential of unobserved confounding, I employ instrumental variable regression using the “*fixest”* package in R. The estimation involves the following two-stage least squares models:

**1. First Stage:**


(3) $$\begin{array}{l} \log\left(visitors\right)=\alpha_{0}+\alpha_{1}\;*\;CLOSURE+\Delta_{i}+\nabla_{j}+\epsilon \; \end{array}$$

The first-stage regression predicts the number of visitors using the street closure variable as the instrument.

**2. Second Stage:**


(4) $$CRIME_{ij}=\beta_{0}+\beta_{1}\;*\;\log(\hat{visit}ors)+\Delta_{i}+\nabla_{j}+u\;$$

The second-stage regression predicts crime rates using the predicted values of visitors from the first stage. The estimate of β_1_ represents the instrumental variable estimate of the causal effect of the number of visitors on neighborhood crime rates.

This methodological approach allows me to address potential endogeneity in the relationship between visitors and crime rates, providing a more robust estimate of the causal effect relative to traditional methods that operate under the “no unobserved confounding” assumption. Notably, while the IV regression relies on arguably weaker assumptions than the two-way fixed effects models, it may be (and often is) a less efficient estimator—so it is not inherently preferred.

## Results

Table 1 presents the results of the two-way fixed effects models. These models include the logged count of visitors on that given day to that given census block group as the sole predictor variable, in addition to census block group-fixed effects and day-fixed effects. The results reveal a significant and positive effect of visitors for six of the seven outcomes under study. The sole outcome for which logged visitors fails to be a statistically significant predictor for is impaired driving. Since the outcome is coded dichotomously, the coefficients can be interpreted in terms of differences in the probability that one or more crimes (of the specified type) occur that day. Substantively, a 10% increase in the number of visitors to a neighborhood is associated with a 0.2% increase in the probability that a crime occurs in that neighborhood on that given day.

**Table 1:** Two-way fixed effects models predicting crime probabilities.

	**Petit larceny**	**Assault**	**Felony assault**	**Murder**	**Impaired driving**	**Traffic violations**	**All crimes**
Logged visitors	0.005^***^	0.005^***^	0.001^***^	0.000^*^	0.000	0.001^***^	0.021^***^
	(0.001)	(0.000)	(0.000)	(0.000)	(0.000)	(0.000)	(0.001)
*N*	2,351,058	2,351,058	2,351,058	2,351,058	2,351,058	2,351,058	2,351,058
BIC	−1,550,230.255	−2,527,706.523	−4,614,613.381	−14,486,412.519	−8,088,528.957	−7,050,393.862	1,581,359.590
Adj. R^2^	0.102	0.027	0.015	0.000	0.011	0.009	0.139

^***^p < 0.001; ^**^p < 0.01; ^*^p < 0.05.

Table 2 presents an analogous set of models where the demeaned logged count of visitors is substituted for the logged count of visitors. I demean the logged count of visitors by subtracting a census block group's average logged daily count of visitors for all of 2019 from the true logged count of visitors, and then rescaling by the standard deviation for that census block group's logged daily count of visitors for all of 2019. The results here are strikingly similar to Table 1. The coefficients can be interpreted in terms of the effect of a one standard deviation change in the logged count of visitors on the probability of one or more crimes (of a specific type) occurring. For example, a one standard deviation increase in the number of visitors to a neighborhood is associated with a 0.6% increase in the probability that a crime occurs in that neighborhood on that given day.

**Table 2:** Two-way fixed effects models predicting crime probabilities with demeaned predictor variable.

	**Petit larceny**	**Assault**	**Felony assault**	**Murder**	**Impaired driving**	**Traffic violations**	**All crimes**
Demeaned logged visitors	0.001^***^	0.001^***^	0.000^***^	0.000^***^	0.000	0.000^***^	0.006^***^
	(0.000)	(0.000)	(0.000)	(0.000)	(0.000)	(0.000)	(0.000)
*N*	2,351,058	2,351,058	2,351,058	2,351,058	2,351,058	2,351,058	2,351,058
BIC	−1,550,225.473	−2,527,743.427	−4,614,624.318	−14,486,422.795	−8,088,528.578	−7,050,401.386	1,581,254.768
Adj. R^2^	0.102	0.027	0.015	0.000	0.011	0.009	0.139

^***^p < 0.001; ^**^p < 0.01; ^*^p < 0.05.

Table 3 presents the results of the instrumental variable regression. The first-stage F-statistic is 58.0, indicating that the IV is a strong instrument. The models indicate, however, that the logged count of visitors has no statistically significant effect on any of the forms of crime under study. Notably, the standard errors are incredibly large, indicating a large degree of uncertainty around the point estimates. Results from the Wu-Hausman test indicate for each of the seven types of crime analyzed, the OLS estimator is consistent and that the regressor is exogenous. This potentially indicates that IV is not necessary.

**Table 3:** Instrumental Variable Regression Results.

	**Petit larceny**	**Assault**	**Felony assault**	**Murder**	**Impaired driving**	**Traffic violations**	**All crimes**
Logged visitors	0.014	−0.006	0.001	−0.005	0.030	−0.039	−0.149
	(0.125)	(0.092)	(0.054)	(0.007)	(0.031)	(0.040)	(0.235)
*N*	2,351,058	2,351,058	2,351,058	2,351,058	2,351,058	2,351,058	2,351,058
BIC	−1,549,918.125	−2,527,058.304	−4,614,607.004	−14,466,915.659	−8,032,973.601	−6,989,592.846	1,610,133.877
Adj. R^2^	0.102	0.027	0.015	−0.008	−0.012	−0.017	0.128

^***^p < 0.001; ^**^p < 0.01; ^*^p < 0.05.

## Discussion

In this paper, I have tested two approaches for estimating the causal effect of neighborhood visitor counts on crime rates. The first approach, two-way fixed effects, indicates a significant and positive relationship between visitor volume and crime, across a variety of crime outcomes. The second approach, an IV regression leveraging street closures as an IV, suggests no statistically significant effect. Notably, however, large standard errors on the IV regression suggest substantial uncertainty in the effect estimates generated via this method. Results from the Wu-Hausman test indicate that two-way fixed effects may serve as a better identification strategy, therefore providing suggestive evidence of a positive causal effect. Ultimately, the IV method offers a new identification strategy for estimating the causal effect of neighborhood visitor volume, but does have limitations.

Overall, the results of the two-way fixed effect models align with earlier work suggesting that an increased volume of visitors to a neighborhood is associated with higher crime rates. This correlation has been documented across a variety of studies measuring the relationship between ambient population with criminal activity (Rummens et al., 2021; Hanaoka, 2016). While it is unclear if insignificant IV estimates are attributable to inefficient statistical estimation—or a true null effect—the IV effect estimates suggest, at a minimum, that caution should be exercised in interpreting the marginal association of visitors with crime rates as a causal effect. Inconsistencies in the findings between two-way fixed effects and IV regression may indicate that the relationship between visitors and crime is confounded by other attributes at the neighborhood-day-level—for example, social disorder, policing practices, or economic activity. A realistic possibility is that visitors do not contribute directly to crime but are correlated with a larger set of urban activities that influence levels of crime. For example, neighborhoods may attract more visitors on days when there is more economic activity, nightlife, law enforcement, or other phenomena that could influence crime trends independent of the volume of visitors.

There are a few limitations of this analysis to keep in mind when interpreting the results. Two-way fixed effects models have a variety of shortcomings that have been well-documented in past research (Imai and Kim, 2021). While IV regression does not suffer from many flaws of two-way fixed effects, it does have a distinct set of assumptions and subsequent limitations. Street closures could influence crime through channels other than visitor volume. For example, closures may disrupt police patrol routes, thereby reducing the measurement of crime even in the absence of criminal offending rates remaining the same. Additionally, closures may affect local business operations (e.g., deliveries or customer access), or alter routine activity patterns in ways that change crime opportunities independently of visitor flows. Such pathways would violate the exclusion restriction assumption, thereby rendering IV estimates biased. Although the analysis includes neighborhood and day fixed effects to mitigate time-invariant and temporal confounding, these adjustments uniquely cannot account for these alternative mechanisms that directly violate the exclusion restriction assumption. Future research could strengthen causal claims by employing complementary instruments, leveraging natural experiments, or applying formal sensitivity analyses to assess how violations of the exclusion restriction might influence estimated effects. One other limitation of the IV approach used here is that the binary instrument (street closure) can only identify an average linear effect of visitor volume on crime risk. The rigid definition of street closures does not allow for testing potential nonlinear or threshold effects. Future research using continuous instruments could help explore whether crime responses to visitor volume vary at different levels.

Additionally, the interpretation of these results is constrained to the study's context—which is just a single city (New York) and year (2019). Analyses performed in other contexts may enhance the generalizability of these findings. While New York City is the largest city in the United States, therefore offering an ideal site for a large-scale study of this nature, neighborhood heterogeneity in such a large, diverse city can also present challenges. The relationship between visitors and crime may vary between different type of neighborhoods, and may even vary between New York and other urban settings with different tourism, policing, or social contexts. Lastly, the analysis did not distinguish between types of visitors—which may mask meaningful variation in the causal effect of visitor volume on crime rates. For example, the volume of tourists, as opposed to commuters, or even as opposed to local shoppers, may present differential effects on crime, which unfortunately cannot be distinguished in this data. Future research could complement this work by refining visitor classifications and examining heterogeneity in effects between different visitor types and across neighborhood destination contexts.

Despite these limitations, this study makes a meaningful contribution to the understanding of mobility and crime by presenting two methods for potentially identifying a causal effect of visitor flows on crime, as well as introducing a novel IV approach to address endogeneity concerns. Methodologically, this analysis suggests that while visitor presence and crime rates are correlated, establishing a causal link remains challenging. Future work could build on this study by employing alternative identification strategies, such as difference-in-difference designs or natural experiments, to further interrogate the causal impact of visitor presence on crime. Additionally, given the growing availability of granular mobility data, future research could explore how different forms of mobility—such as non-residential worker flows, nightlife activity, or transit ridership—interact with neighborhood crime patterns. More broadly, this study underscores the importance of carefully considering endogeneity when studying urban crime dynamics and highlights the need for continued causal inference methodological innovation in this area.

These findings also carry important policy implications, especially for urban planners and law enforcement agencies. Given the substantial uncertainty in these findings, policymakers should exercise great caution in relying on visitor flow data for predictive policing or resource allocation. The risk of acting on potentially spurious associations can perpetuate inequitable policing practices and reinforce existing spatial patterns of surveillance. Instead, urban planners might focus on broader social and environmental design strategies to enhance safety without intensifying policing. Ethical considerations call for greater transparency about the limits of predictive models, resisting their use as justification for punitive interventions, and ensuring that big data is not weaponized in ways that harm marginalized communities.

## Data Availability

The data analyzed in this study is subject to the following licenses/restrictions: The data underlying these findings cannot be shared due to the data sharing agreement under which the data was originally accessed. Requests to access these datasets should be directed to safegraph.com.
